# A Subjective and Objective Assessment of Combined Methods of Applying Chemical Peels and Microneedling in Antiaging Treatments

**DOI:** 10.3390/jcm12051869

**Published:** 2023-02-27

**Authors:** Agata Markiewicz-Tomczyk, Elzbieta Budzisz, Anna Erkiert-Polguj

**Affiliations:** 1 Department of Cosmetic Raw Materials Chemistry, Faculty of Pharmacy, Medical University of Lodz, 90-151 Lodz, Poland; 2 Department of Cosmetology and Aesthetic Dermatology, Faculty of Pharmacy, Medical University of Lodz, 90-151 Lodz, Poland

**Keywords:** antiaging therapy, azelaic acid, claim substantiation, hydration, microneedling, skin repair, vitamin C

## Abstract

Combined methods of applying chemical peels and antioxidants could be an option for skin rejuvenation with no down-time. The penetration of active substances can be enhanced by microneedle mesotherapy. The study was conducted on a group of 20 female volunteers, aged 40–65 years. All volunteers received a series of eight treatments performed every seven days. The whole face was first treated with azelaic acid; following this, the right side received a 40% solution of vitamin C and the left side 10% vitamin C with microneedling. Hydration and skin elasticity were markedly improved, with better results observed on the microneedling side. Melanin and erythema index decreased. No significant side effects were seen. The combination of active ingredients and delivery techniques have great potential to enhance the effectiveness of cosmetic preparations, probably by multidirectional ways of action. In our study, we demonstrated that both 20% azelaic acid + 40% vitamin C treatment and 20% azelaic acid + 10% vitamin C + microneedle mesotherapy efficiently improved the assessed parameters of aging skin. However, the use of microneedling mesotherapy as a means of direct delivery of active compound to the dermis enhanced the effectiveness of the studied preparation.

## 1. Introduction

Skin aging is a complex, multifactorial process which starts to gradually appear in the third decade of life onwards and accelerates with age [1]. Such aging occurs earlier than other tissues, possibly as a result of exposure to environmental stressors and hazards. In general, the characteristic features of skin aging involve a loss of elasticity, appearance of wrinkles, and rough-textured appearance [2]. Such changes are associated with phenotypic alterations of cutaneous cells and structural and functional modifications occurring in extracellular matrix components, including elastin, collagens, and proteoglycans. These components are necessary for skin elasticity, tensile strength, and hydration [3]. Skin aging is mirrored by a reduction in collagen I content and the fragmentation of collagen fibrils as well as the accumulation of amorphous elastin material (elastosis) [4].

Aging-related alterations in skin appearance can negatively affect self-esteem and can result in substantial psychosocial burden [1]. Therefore, products that can help to delay this continuous process are of high importance in everyday life. A wide variety of cosmetic and medical antiaging products have been developed and introduced to the market; however, their efficiency is variable. Despite assurances from manufacturers, few topical antiaging components are able to effectively penetrate into the dermis; therefore, various resurfacing techniques, such as chemical peeling, mesotherapy, filler injections, and laser/radiofrequency tightening, are used to improve the delivery of active substances.

A number of antioxidant substances can be used to fight the signs of aging by neutralizing reactive oxygen species (ROS) that damage DNA, limiting collagen-degrading matrix metalloproteinase (MMP) production and inhibiting inflammation by the NF-κB pathway [2,5]. These can be nonenzymatic, such as vitamin C and E, or enzymatic, such as glutathione peroxidase, catalase, superoxide dismutase, and coenzyme Q10. Vitamin C (ascorbic acid) is a well-known antioxidant which is capable of neutralizing and removing oxidants [6]. This property seems to be of special importance in the epidermis, where vitamin C is concentrated within the skin [7]. It also serves as a cofactor for the enzymes necessary for post-translational processing in the biosynthesis of collagen Types I and III [8,9] and stabilizes the tertiary structure of the collagen molecule [7,10,11,12]. Vitamin C has also been found to enhance the proliferation and migration of dermal fibroblasts and differentiation of rat epidermal keratinocytes in an organotypic culture model, thus significantly improving the ultrastructural organization of the stratum corneum and barrier function [10,13,14]. In addition, vitamin C derivatives appear to reduce the synthesis of melanin by disturbing the action of tyrosinase, the rate-limiting enzyme in melanogenesis [7].

Azelaic acid, a dicarboxylic acid, has multifaceted properties. It prevents discoloration by inhibiting the activity of tyrosinase and evens out the structure and color of the skin. It exerts its antiaging properties by inhibiting neutrophil activity, thus preventing free radical production, and scavenges ROS [15]. Due to its strong antibacterial and anti-inflammatory properties, it is successfully used in antiacne therapies, as well as in melasma and rosacea [16].

The present study uses microneedle mesotherapy. This is a technique that uses a dense arrangement of microinjections to enhance the penetration of active ingredients into deeper layers of the skin, i.e., it comes into contact with the dermal fibroblast cells. The procedure has been found to stimulate tissue regeneration [17].

The aim of this study was to assess the antiaging properties of methods of applying chemical peels used in combination.

## 2. Materials and Methods

### 2.1. Participants

The study was conducted during winter on a group of 20 female volunteers, aged 40–65 years. All patients were informed about the procedures and of any possible side effects, including itching, shedding, redness, burning, and excessive skin sensitivity to external factors. In case of any undesirable symptoms, patients were required to immediately inform the cosmetologist. During the study, and one month after its end, participants were recommended to use creams with SPF 50 in the mornings and during the day, asked not to use any other cosmetic treatments, not to change skin care habits, and not to consume coffee, hot drinks or spicy food at least two hours before the measurements. All patients indicated compliance with these recommendations.

Written informed consent to take part was obtained from each patient before the start of the procedure. The study was conducted in accordance with the Declaration of Helsinki and the recommendations and guidelines of Cosmetics Europe. Moreover, the study was approved by the Bioethics Committee of the Medical University of Lodz (no. RNN/281/16/KE 2017).

### 2.2. Procedures

Treatments were performed every seven days, for a total of eight treatments using the split face method. Before the treatment, make-up was removed from the whole face, and the skin was carefully cleaned with a prepeel product containing 2% salicylic acid. Following this, 20% azelaic acid (pH 2.7) was applied to the entire face for 10 min.

On the right side of the face, without removing the azelaic acid, 40% vitamin C (pH 1.6), stabilized with a novel complex of ferulic and lactobionic acids, was applied for 15–20 min, depending on the skin reaction. After this time, the ascorbic acid was washed off.

**Figure 1:**
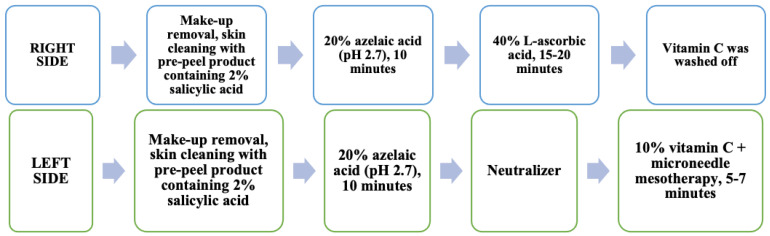
Summary of the method of study products application.

On the left side of the face, after the 10 min azelaic acid application, a neutralizer was used to diminish the effects of acids on skin and eradicate any unpleasant feeling of burning. This was followed by the use of 10% vitamin C and microneedle mesotherapy with a derma roller (0.5 mm) for 5–7 min (Figure 1). A DNS Bielenda 0.5 mm roller equipped with 192 stainless steel needles was used in this study.

### 2.3. Measurement of Skin Parameters

The first skin measurement was performed before the series of treatments (T0), then after eight sessions of treatments (T1—8 weeks), and again four weeks after the end of the treatment (T2—12 weeks). Each measurement of the skin parameters was carried out in a special room with a constant temperature (T = 19–21 °C) and air humidity 30–50%; all participants were allowed to acclimatize for at least 20 min in a waiting room with the required temperature and air humidity. The skin was cleansed at least four hours before the examination. Measurements were taken on the cheeks and forehead, on both the right and left sides of the face, with the use of Multi Probe Adapter Systems (Courage + Khazaka electronic GmbH, Köln, Germany). The following parameters were assessed: hydration (Corneometr), skin tone (Mexametr), and elasticity (Cutometr). All measurements were performed three times in each location and the mean value was used for analysis. Skin tone was measured preferably in the area of discoloration or telangiectasia.

Skin properties were evaluated based on two parameters: R2 (total elasticity of the skin) and R7 (index of immediate contraction after complete deformation). To make an objective assessment of the effects during the treatments, photographs were also taken in five positions with the Fotomedicus (ELFO) system. After the series of treatments, the participants were asked to complete a questionnaire allowing a subjective assessment of facial skin. The questionnaire contained 15 questions concerning subjective opinion of participants on the observed changes in skin appearance, including the reduction of wrinkles visibility, improvement of skin hydration, firmness, elasticity, and tone, as well as decrease in skin redness, hypersensitivity, and feeling of tension. The answers were definitely yes, yes, do not know, or no.

### 2.4. Statistical Analysis

Continuous variables were tested for normality with the Shapiro–Wilk test. Parameters with a normal distribution were described using mean and standard deviation (mean ± SD). Non-normally distributed variables were expressed as median and interquartile range: median (25%; 75%). Changes in normally distributed skin parameters over time were evaluated using one-way repeated measures ANOVA (with either Greenhouse–Geisser or Huynh–Feldt correction for sphericity when appropriate), followed by Bonferroni’s post hoc test. Differences in the percentage changes of skin parameters compared to baseline values (([x(t_1_) − x(t_0_)]/x(t_0_)) × 100) between different treatments and skin locations were analysed with the Mann–Whitney test. *p* values of less than 0.05 were considered to be statistically significant.

## 3. Results

The study included 20 female volunteers aged 40–65 years. All participants had skin that showed signs of aging, such as hyperpigmentation, erythema, telangiectasia, wrinkles, and local feelings of dryness.

Both types of treatment yielded significant improvements in all assessed skin parameters. Both 20% azelaic acid + 40% vitamin C treatment and 20% azelaic acid + 10% vitamin C + microneedle mesotherapy efficiently improved skin tone (Table 1).

**Figure 2:**
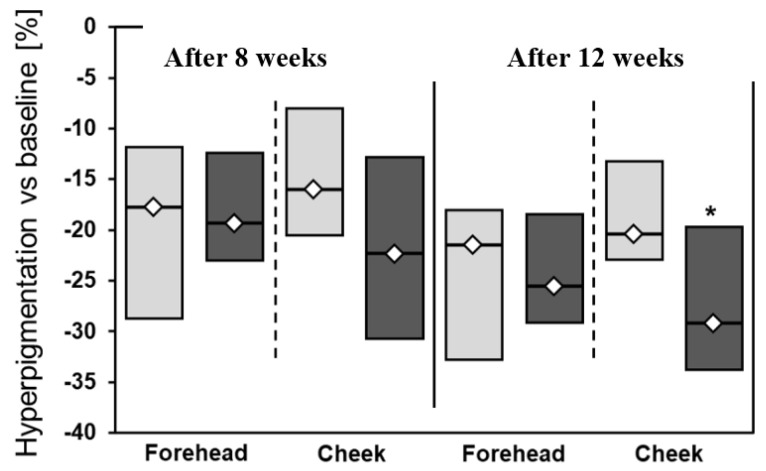
Percentage changes in hyperpigmentation after 8 and 12 weeks of treatment. * *p* < 0.05; light grey: right side of the face; dark grey: left side of the face. Mann-Whitney test was used for calculations.

**Figure 3:**
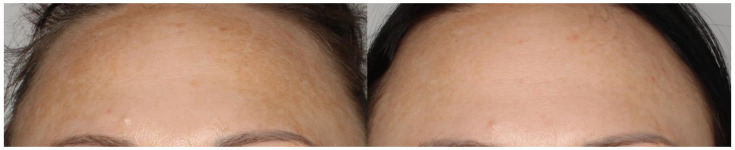
The reduction of skin hyperpigmentation.

**Figure 4:**
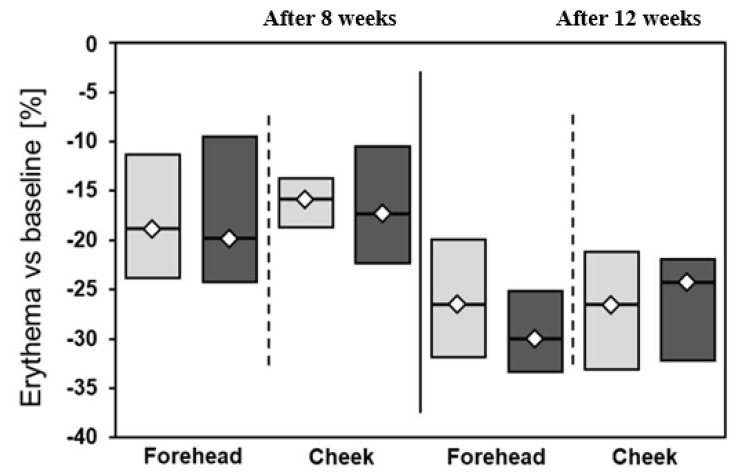
Percentage changes in erythema after 8 and 12 weeks of treatment. Light grey: right side of the face; dark grey: left side of the face; Mann–Whitney test was used for calculations.

A slightly greater decrease in pigmentation (I measurement vs. III measurement) was observed on the left side of the face (25.5% on forehead and 29.7% on cheek) in comparison to the right side (21.4% on forehead and 20.3% on cheek) (Figure 1). Both applied treatments were associated with significant improvement of erythema. The combination of 20% azelaic acid with 10% vitamin C and microneedle mesotherapy was also associated with slightly better, but insignificant, effects related to erythema reduction compared to 20% azelaic acid + 40% vitamin C (Table 2, Figure 2, Figure 3 and Figure 4).

**Figure 5:**
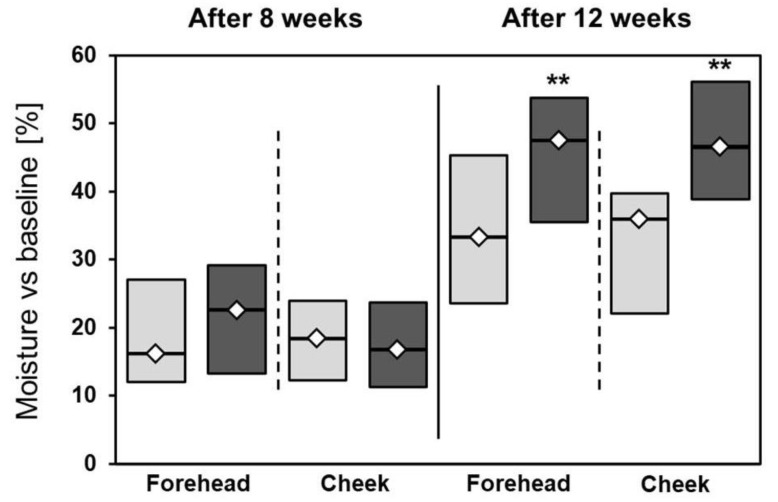
Percentage changes in hydration after 8 and 12 weeks of treatment. ** *p* < 0.01; light grey: right side of the face; dark grey: left side of the face; Mann–Whitney test was used for calculations.

The therapy markedly improved skin hydration of both sides of the face (Table 3); however, markedly better hydration was demonstrated on the left side compared to the right. Compared to baseline (i.e., measurement III vs. measurement I), these values were 47.5% (35.5; 53.8) vs. 33.3% (23.6; 45.3) on the forehead (*p* = 0.002), and 46.4% (38.9; 56.1) vs. 36.0% (22.1; 39.7) on the cheek (*p* = 0.001) (Figure 5).

**Figure 6:**
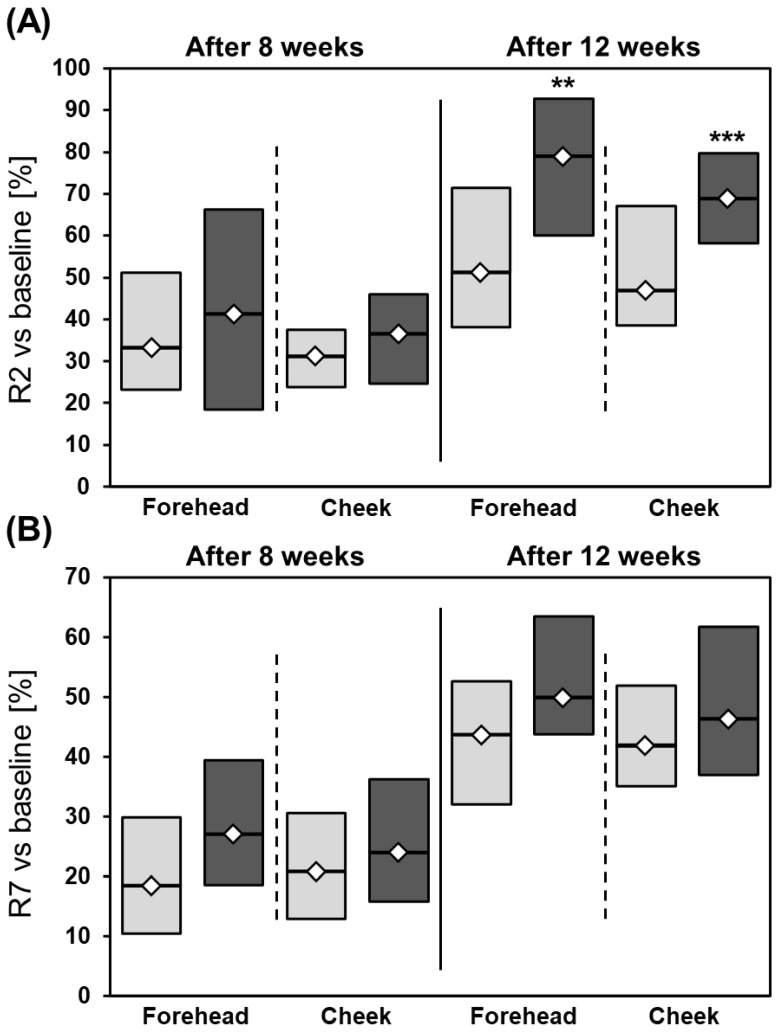
Percentage changes in R2 (**A**) and R7 (**B**) after 8 and 12 weeks of treatment. ** *p* < 0.01, *** *p* < 0.0001; light grey: right side of the face; dark grey: left side of the face; Mann–Whitney test was used for calculations.

The elasticity of skin was found to be significantly reduced at the end of the treatment in all participants (Table 4). R2 was found to increase by 79.0% on the forehead and 69.0% on the cheek after combined use of 20% azelaic acid + 10% vitamin C and mesotherapy. However, much-less-pronounced effects were observed on the right side (forehead: 51.2%, cheek: 46.9%). The differences between both sides and both used methods were statistically significant (Figure 6).

The R7 values increased by nearly 50% on the left side (forehead: 49.8%, cheek: 46.3%) and by more than 40% on the right side (forehead: 43.7%, cheek: 42.0%) in all participants (Table 5, Figure 6).

**Figure 7:**
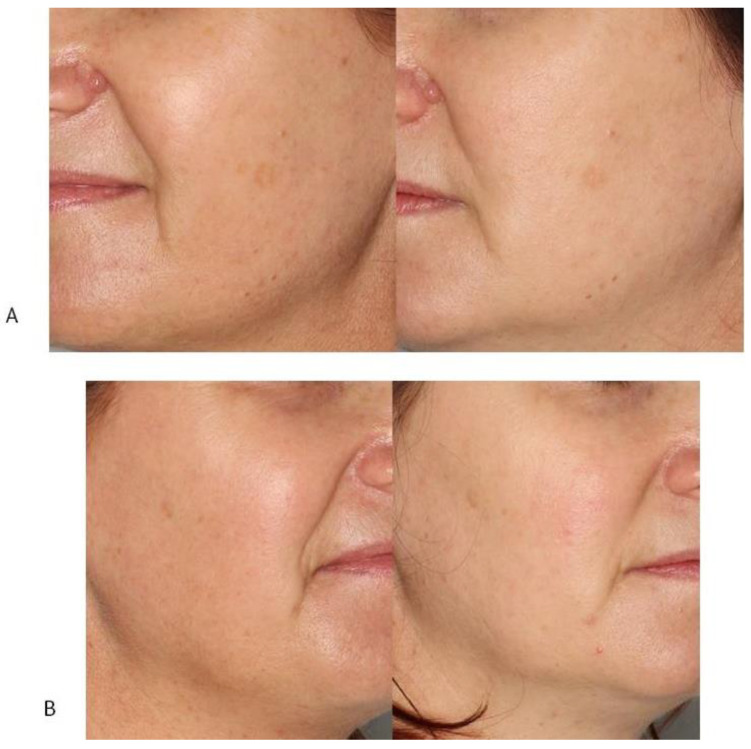
The overall improvement of skin condition on the left (**A**) and right (**B**) sides.

The overall improvement of skin condition is shown in Figure 7.

**Figure 8:**
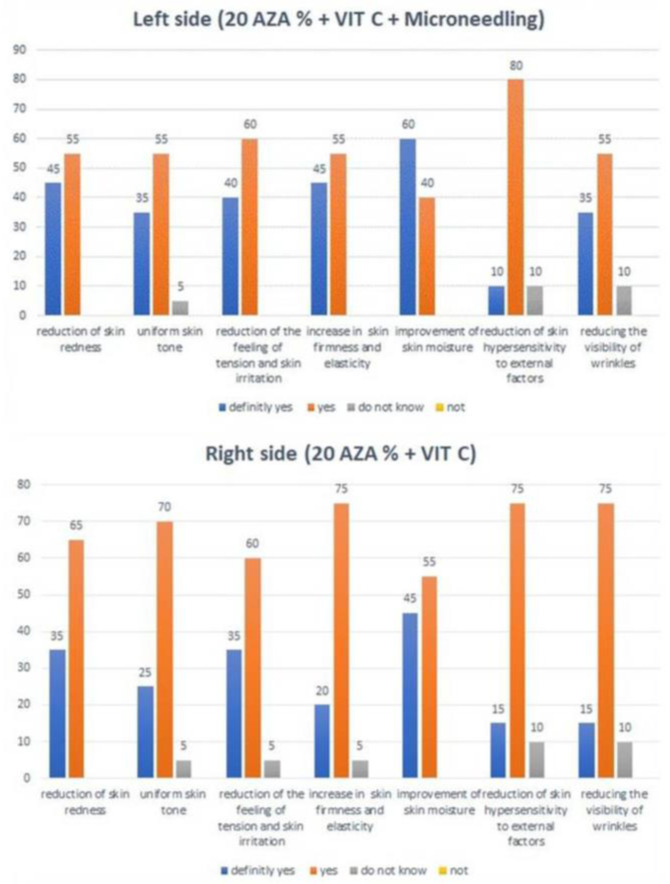
The subjective opinion of the participants regarding the improvement of skin appearance after the completion of treatment.

The questionnaire results indicated that a great majority of participants were satisfied with the observed effects. They reported improved skin hydration, greater skin firmness and elasticity, and reduced redness and wrinkles. In this subjective assessment, all the evaluated skin features were improved to a slightly larger extent after the use of 20% azelaic acid + 10% vitamin C + microneedle mesotherapy compared to the other treatment (Figure 8).

Side effects of peels and mesotherapy may include redness, itching, burning, hypersensitivity to external factors, hyperpigmentation; however, in this study, no significant side effects were observed. It must be mentioned that the safety of the procedure was very important to us.

## 4. Discussion

The process of skin aging is associated with its gradual deterioration. This has been attributed to both intrinsic and extrinsic skin aging processes. Intrinsic aging is a natural progressive physiological process leading to the formation of fine wrinkles, skin thinning and dryness, as well as gradual dermal atrophy. In turn, extrinsic aging results from the impact of external environment factors, including exposure to ultraviolet (UV) light, infrared radiation, environmental pollution, tobacco smoking, malnutrition, and psychological stress; this leads to the loss of skin elasticity, greater laxity, and the formation of coarse wrinkles [1,2]. Extrinsic factors also appear to deplete antioxidant levels in skin and increase reactive oxygen species (ROS) levels [5].

A recent theory suggests that aging could be associated with impaired redox-stress response capacity (RRC) [18]; aging is associated with a gradual worsening of the endogenous antioxidant system and thus the reduced capacity of elderly skin [19]. Therefore, the application of exogenous antioxidants should boost antioxidant capacity and diminish UV-induced skin photodamage and photoaging.

It is estimated that premature skin aging affects up to 83% of adults under the age of 55 [1,20]. Since having a young and attractive appearance may exert a positive influence on social behavior and mood, there is a growing interest in therapies that help to alleviate or reduce the effects of aging [2]. Antioxidant-based therapy, using vitamin C, vitamin E, or polyphenols, may improve the resistance to ROS-mediated oxidative damage, decrease inflammation, and slow skin aging [21].

The present study assessed the effectiveness of two types of antiaging therapy in middle-aged women whose skin showed signs of aging such as hyperpigmentation, erythema, telangiectasia, wrinkles, and local feeling of dryness. The findings indicate significant improvements in all assessed skin parameters after both types of treatment: both 20% azelaic acid + 40% vitamin C treatment and 20% azelaic acid + 10% vitamin C + microneedle mesotherapy efficiently improved skin hyperpigmentation as well as erythema. Other studies have also found azelaic acid to have a beneficial impact on hyperpigmentation by inhibiting tyrosinase and exerting an antiproliferative effect on the melanogenesis pathway [22,23]. Mazurek et al. indicated that 5–20% azelaic acid combined with phytic acid, ferulic acid, 4N-butyl resorcinol, or mandelic acid is most effective in lightening hyperpigmentation; also, vitamin C application was found to reduce the degree of pigmentation [24]. A clinical trial studying the effectiveness of a preparation containing 25% l-ascorbic acid with a chemical penetration enhancer demonstrated marked reduction in pigmentation severity in patients with melasma [25].

Vitamin C has been found to diminish the level of skin erythema by affecting blood microcirculation. Additionally, the addition of ferulic acid not only increases the stability of vitamin C preparation, but also improves its skin-lightening properties, since ferulic acid hampers tyrosinase activity [26,27]. Kameyama et al. observed that topical application of ascorbic acid derivative (magnesium-ascorbyl-2-phosphate, MAP) effectively lightened the skin of patients with hyperpigmentation disorders, such as melasma or solar lentigines [28].

In this study, both types of treatment markedly improved skin hydration; however, a much more pronounced effect was observed on the side of the face receiving the microneedle mesotherapy. The improvement in hydration could be associated with the use of lactobionic acid (LA) since it was found that LA peels could enhance skin hydration and limit transepidermal water loss [29]. Additionally, it has been suggested that mesotherapy may help intensify the level of skin hydration [30]. Another study found the use of azelaic acid to diminish the effects of aging, particularly dryness, via the regulation of sebum secretion [16].

Skin elasticity, assessed based on R2 and R7, was also found to be significantly ameliorated by both therapies. However, significantly better effects were observed on the left side of the face, i.e., the side treated with mesotherapy. On this side, the R2 value improved by 79.0% on the forehead and 69.0% on the cheek. R2 indicates the ability of the skin to return to its original position after deformation and reflects the function of the elastic fibers of the skin [31]. Additionally, parameter R7, showing the ability of the skin to recover after deformation, was markedly improved in comparison with baseline; however, again, the percentage change was higher after the use of 20% azelaic acid + vitamin C + microneedle mesotherapy. Thus, it seems that the use of mesotherapy enhances the antiaging effects of the used antioxidants, possibly by enabling their delivery to deeper skin layers.

The beneficial effects of mesotherapy on aging skin have been confirmed in other studies. Baspeyras et al. demonstrated that HA-based mesotherapy significantly and sustainably improved skin elasticity. The author notes that this minimally invasive procedure supports the creation of an optimal physiological environment for fibroblasts and promotes their ability to produce the extracellular matrix. Such conditions are conducive to enhanced collagen and elastin synthesis [32,33]. Fibroblasts produce substances that are vital for maintaining a youthful skin condition [32]. The direct delivery of active substances using microinjections has been found to stimulate elastic and collagen fibers production [30], as the healing process promotes skin regeneration and repair. It seems that the great improvement in skin elasticity achieved by the present treatments, especially the one incorporating microneedling mesotherapy, is related not only to effects of vitamin C but also to the method of application.

Aged and photoaged skin is characterized by flattened epidermal–dermal junctions and the disappearance of papillary projections and larger corneocytes [34]. Previous studies suggest that topical application of vitamin C may partly restore the appropriate structure of the epidermal–dermal junction and papillary dermis. Sauermann et al. [35] report that the application of 3% vitamin C on the forearm of women improved the density of dermal papillae, probably via angiogenesis, suggesting that this compound can partially correct aging-related regressive structural changes. A double-blind, randomized trial revealed that topical application of 5% vitamin C on photoaged skin was associated with marked intensification of the density of skin microrelief as well as a reduction of the deep furrows [36]. Such treatment was not only effective but also well-tolerated.

Other studies have reported a decrease in the appearance of facial fine lines/wrinkles [6,37]. The application of ascorbic acid markedly diminished oxidative stress in the skin, improved epidermal–dermal microstructure, and decreased fine lines and wrinkles in aged skin [37]. Another study found that vitamin C treatment resulted in lowered photoaging scores, increased hydration, and increased Grenz zone collagen levels; in addition, higher levels of type I collagen mRNA were revealed by staining [38]. Additionally, chemical peelings which remove damaged facial skin in a controlled manner improve the smoothness and texture of skin [39]. Additionally, azelaic acid is known to inhibit the production of free radicals by neutrophils, which suggests that it may have a beneficial role in antiaging therapies.

It seems that all the ingredients used in the combined therapy herein have antiaging effects. In addition, the objective efficiency of the applied treatment was also confirmed by the results of questionnaire, which clearly showed that a great majority of participants was satisfied with the observed effects. They observed improved skin hydration, firmness, and elasticity, as well as the reduction of redness and wrinkles. It is worth underlying that the observed effects were long lasting, since the measured values continued to improve even after four weeks from the end of the treatment. This finding may suggest that the applied treatment triggered long-lasting skin remodeling.

Our study has some limitations. Firstly, the study included a small number of participants. Moreover, all participants were females and they belonged to the white race (Caucasian race). Therefore, the obtained results cannot be extrapolated to patients of other races.

## 5. Conclusions

The choice of active ingredient and delivery technique has great potential to enhance the effectiveness of cosmetic preparations. The method used in this study, based on the combination of two active ingredients and the use of transdermal application, turned out to be both safe and effective. We achieved promising effects, and these could be associated with the multidirectional effects of combination therapy on aging skin. Our findings indicate that both 20% azelaic acid + 40% vitamin C treatment and 20% azelaic acid + 10% vitamin C + microneedle mesotherapy efficiently improved the assessed parameters of aging skin. However, the use of microneedling mesotherapy, as a means of direct delivery of active compound to the dermis, enhanced the effectiveness of the studied preparation.

## Figures and Tables

**Table 1:** Skin tone assessment.

Skin Tone N = 20	Site of Measurement	Time Point			*p*
		Measurement I Baseline (*)	Measurement II 8 Weeks (#)	Measurement III 12 Weeks	
Left side (A + 10%Vit C + M)	Forehead	144.0 ± 17.5	*** 116.6 ± 20.9	*** 108.8 ± 19.2 ###	*p* < 0.0001
	Cheek	133.4 ± 19.7	*** 103.8 ± 24.8	*** 96.5 ± 21.2 ###	*p* < 0.0001
Right side (A + 40% Vit C)	Forehead	143.5 ± 15.0	*** 117.7 ± 22.2	*** 109.8 ± 23.3 #	*p* < 0.0001
	Cheek	130.0 ± 14.3	*** 108.6 ± 19.1	*** 102.9 ± 18.0 ###	*p* < 0.0001

* *p* < 0.05; *** *p* < 0.001 vs. measurement I; # *p* < 0.05; ### *p* < 0.001 vs. measurement II; A: 20% azelaic acid; vit C.: vitamin C; M: microneedle mesotherapy; ANOVA and Bonferroni’s post hoc test were used for calculations.

**Table 2:** Erythema assessment results.

Erythema N = 20	Site of Measurement	Time point			*p*
		Measurement I (*)	Measurement II (#)	Measurement III	
Left side (A + 10%Vit C + M)	Forehead	336.0 ± 44.3	*** 270.5 ± 47.3	*** 234.0 ± 29.2 ###	*p* < 0.0001
	Cheek	354.5 ± 51.7	*** 294.6 ± 50.2	*** 255.3 ± 44.1 ###	*p* < 0.0001
Right side (A + 40% Vit C)	Forehead	331.2 ± 47.8	*** 268.4 ± 47.5	*** 243.0 ± 35.5 ###	*p* < 0.0001
	Cheek	343.6 ± 51.0	*** 285.7 ± 50.2	*** 250.7 ± 46.0 ###	*p* < 0.0001

* *p* < 0.05; *** *p* < 0.001 vs. measurement I; # *p* < 0.05; ### *p* < 0.001 vs. measurement II; A: 20% azelaic acid; vit C.: vitamin C; M: microneedle mesotherapy; ANOVA and Bonferroni’s post hoc test were used for calculations.

**Table 3:** The result of hydration measurement.

Hydration N = 20	Site of Measurement	Time point			*p*
		Measurement I (*)	Measurement II (#)	Measurement III	
Left side (A + 10%Vit C + M)	Forehead	45.7 ± 6.6	*** 56.9 ± 9.1	*** 67.6 ± 7.3 ###	*p* < 0.0001
	Cheek	45.5 ± 4.8	*** 53.1 ± 6.3	*** 66.7 ± 5.1 ###	*p* < 0.0001
Right side (A + 40% Vit C)	Forehead	46.2 ± 6.5	*** 54.4 ± 7.6	*** 61.0 ± 7.4 ###	*p* < 0.0001
	Cheek	45.5 ± 4.7	*** 54.2 ± 6.4	*** 60.6 ± 7.1 ###	*p* < 0.0001

* *p* < 0.05; *** *p* < 0.001 vs. measurement I; # *p* < 0.05; ### *p* < 0.001 vs. measurement II; A: 20% azelaic acid; vit C.: vitamin C; M: microneedle mesotherapy; ANOVA and Bonferroni’s post hoc test were used for calculations.

**Table 4:** The result of R2 measurement.

R2	Site of Measurement	Time Point			*p*
		Measurement I (*)	Measurement II (#)	Measurement III	
Left side (A + 10% Vit C + M)	Forehead	0.440 ± 0.035	*** 0.632 ± 0.112	*** 0.786 ± 0.095 ###	*p* < 0.0001
	Cheek	0.457 ± 0.031	*** 0.616 ± 0.066	*** 0.776 ± 0.044 ###	*p* < 0.0001
Right side (A + 40% Vit C)	Forehead	0.465 ± 0.057	*** 0.626 ± 0.089	*** 0.704 ± 0.052 ###	*p* < 0.0001
	Cheek	0.460 ± 0.048	*** 0.596 ± 0.064	*** 0.685 ± 0.059 ###	*p* < 0.0001

* *p* < 0.05; *** *p* < 0.001 vs. measurement I; # *p* < 0.05; ### *p* < 0.001 vs. measurement II; A—20% azelaic acid; vit C.: vitamin C; M: microneedle mesotherapy; ANOVA and Bonferroni’s post hoc test were used for calculations.

**Table 5:** The result of R7 measurement.

R7	Site of Measurement	Time Point			*P*
		Measurement I (*)	Measurement II (#)	Measurement III	
Left side (A + 10% Vit C + M)	Forehead	0.274 ± 0.039	*** 0.352 ± 0.045	*** 0.420 ± 0.045 ###	*p* < 0.0001
	Cheek	0.282 ± 0.036	*** 0.361 ± 0.046	*** 0.427 ± 0.039 ###	*p* < 0.0001
Right side (A + 40% Vit C)	Forehead	0.294 ± 0.041	*** 0.356 ± 0.056	*** 0.426 ± 0.051 ###	*p* < 0.0001
	Cheek	0.291 ± 0.032	*** 0.354 ± 0.044	*** 0.423 ± 0.036 ###	*p* < 0.0001

* *p* < 0.05; *** *p* < 0.001 vs. measurement I; # *p* < 0.05; ### *p* < 0.001 vs. measurement II; A—20% azelaic acid; vit C.: vitamin C; M: microneedle mesotherapy; ANOVA and Bonferroni’s post hoc test were used for calculations.

## Data Availability

Data are available on request.
